# Causal effect of smoking on DNA methylation in peripheral blood: a twin and family study

**DOI:** 10.1186/s13148-018-0452-9

**Published:** 2018-02-09

**Authors:** Shuai Li, Ee Ming Wong, Minh Bui, Tuong L. Nguyen, Ji-Hoon Eric Joo, Jennifer Stone, Gillian S. Dite, Graham G. Giles, Richard Saffery, Melissa C. Southey, John L. Hopper

**Affiliations:** 1 Centre for Epidemiology and Biostatistics, Melbourne School of Population and Global Health, University of Melbourne, Parkville, Victoria Australia; 2 Genetic Epidemiology Laboratory, Department of Pathology, University of Melbourne, Parkville, Victoria Australia; 3 Precision Medicine, School of Clinical Sciences at Monash Health, Monash University, Clayton, Victoria Australia; 4 Centre for Genetic Origins of Health and Disease, Curtin University and the University of Western Australia, Perth, Western Australia Australia; 5 Cancer Epidemiology and Intelligence Division, Cancer Council Victoria, Melbourne, Victoria Australia; 6 Murdoch Children’s Research Institute, Royal Children’s Hospital, Parkville, Victoria Australia; 7 Department of Paediatrics, University of Melbourne, Parkville, Victoria Australia

**Keywords:** DNA methylation, Smoking, Epigenome-wide association study, Causal inference, Family study

## Abstract

**Background:**

Smoking has been reported to be associated with peripheral blood DNA methylation, but the causal aspects of the association have rarely been investigated. We aimed to investigate the association and underlying causation between smoking and blood methylation.

**Methods:**

The methylation profile of DNA from the peripheral blood, collected as dried blood spots stored on Guthrie cards, was measured for 479 Australian women including 66 monozygotic twin pairs, 66 dizygotic twin pairs, and 215 sisters of twins from 130 twin families using the Infinium HumanMethylation450K BeadChip array. Linear regression was used to estimate associations between methylation at ~ 410,000 cytosine-guanine dinucleotides (CpGs) and smoking status. A regression-based methodology for twins, Inference about Causation through Examination of Familial Confounding (ICE FALCON), was used to assess putative causation.

**Results:**

At a 5% false discovery rate, 39 CpGs located at 27 loci, including previously reported *AHRR*, *F2RL3*, *2q37.1* and *6p21.33*, were found to be differentially methylated across never, former and current smokers. For all 39 CpG sites, current smokers had the lowest methylation level. Our study provides the first replication for two previously reported CpG sites, cg06226150 (*SLC2A4RG*) and cg21733098 (*12q24.32*). From the ICE FALCON analysis with smoking status as the predictor and methylation score as the outcome, a woman’s methylation score was associated with her co-twin’s smoking status, and the association attenuated towards the null conditioning on her own smoking status, consistent with smoking status causing changes in methylation. To the contrary, using methylation score as the predictor and smoking status as the outcome, a woman’s smoking status was not associated with her co-twin’s methylation score, consistent with changes in methylation not causing smoking status.

**Conclusions:**

For middle-aged women, peripheral blood DNA methylation at several genomic locations is associated with smoking. Our study suggests that smoking has a causal effect on peripheral blood DNA methylation, but not vice versa.

**Electronic supplementary material:**

The online version of this article (10.1186/s13148-018-0452-9) contains supplementary material, which is available to authorized users.

## Background

Epigenetics is a mechanism modifying gene expression without changing underlying DNA sequence. DNA methylation, a phenomenon that typically a methyl group (-CH3) is added to a cytosine-guanine dinucleotide (CpG) at which the cytosine is converted to a 5-methylcytosine, has been proposed to play a role in the aetiology of complex traits and diseases [1, 2].

At least 21 epigenome-wide association studies (EWASs) have reported that methylation in the blood of adults at a great many CpGs is associated with smoking status [3–23]. A recent, and the largest meta-analysis so far, reported 18,760 CpGs annotated to 7201 genes, which account for approximately one third of the known human genes, were differentially methylated between 2433 current smokers and 6956 never smokers [11]. Associations for several loci, such as *AHRR*, *F2RL3*, *GPR15*, *GFI1*, *2q37.1* and *6p21.33*, have been consistently reported, and a systematic review published in 2015 found that associations for 62 CpGs had been reported at least three times [24]. Apart from smoking status, other smoking exposures such as cumulative smoking [3, 4, 8–12, 16–18, 20, 22] and years since quitting [4, 9–12, 15, 16, 19, 20, 22] have also been found to be associated with blood DNA methylation.

Most of the reported associations are from cross-sectional designs; thus, the causal nature of the association, i.e. whether DNA methylation has a causal effect on smoking or vice versa, is unknown. There is also a possibility that cross-sectional epigenetic associations are due to familial confounding [25]. Studies have suggested that smoking-related blood DNA methylation mediates the effects of smoking on lung cancer [26, 27], death [28], leukocyte telomere length [29], and subclinical atherosclerosis [30]. These studies assume that smoking has a causal effect on methylation without evidence of causality. To the best of our knowledge, the only causal evidence comes from a study using a two-step Mendelian randomisation (MR) approach to investigate the mediating role of methylation between smoking and inflammation [31]. This study found that smoking had a causal effect on methylation at CpGs located at *F2RL3* and *GPR15* genes.

In this study, we aimed to investigate association between smoking and blood DNA methylation, to replicate associations previously reported and to investigate putative causal nature of the association using regression methods for related individuals.

## Methods

### Study sample

The sample comprised women from the Australian Mammographic Density Twins and Sisters Study [32]. A total of 479 women including 66 monozygotic twin pairs, 66 dizygotic twin pairs and 215 sisters from 130 families were selected [33].

### Smoking data collection

A telephone-administered questionnaire was used to collect participants’ self-reported information on smoking. Participants were asked the question ‘Have you ever smoked at least one cigarette per day for 3 months or longer?’ Participants who answered ‘No’ were classified as never smokers, and the rest ever smokers. Ever smokers were further questioned for age at starting smoking, the average number of cigarettes smoked per day, and age at stopping smoking, if any. Ever smokers who had stopped smoking before the interview were classified as former smokers, and the rest current smokers.

### DNA methylation data

DNA was extracted from dried blood spots stored on Guthrie cards using a method previously described [34]. Methylation was measured using the Infinium HumanMethylation450K BeadChip array. Raw intensity data were processed by Bioconductor *minfi* package [35], which included normalisation of data using Illumina’s reference factor-based normalisation methods (*preprocessIllumina*) and subset-quantile within array normalisation (*preprocessSWAN*) [36] for type I and II probe bias correction. An empirical Bayes batch-effects removal method *ComBat* [37] was applied to minimise technical variation across batches. Probes with missing values (detection *P* value> 0.01) in one or more samples, with documented SNPs at the target CpG, with beadcount < 3 in more than 5% samples, binding to multiple locations [38] or binding to X chromosome, and the 65 control probes were excluded, leaving 411,219 probes included in the analysis; see Li et al. [33] for more details.

### Epigenome-wide association analysis

We investigated the association using a linear mixed-effects model in which the methylation *M* value, a logit transformation of the percentage of methylation, as the outcome and smoking status (never, former and current smokers) as the predictor. The model was adjusted for age and estimated cell-type proportions [39] as fixed effects and for family and zygosity as random effects, fitted using the *lmer()* function from the R package *lme4* [40]. The likelihood ratio test was used to make inference, that is, a nested model without smoking status was fitted and a *P* value was calculated based on that, twice the difference in the log likelihoods between the full and nested models approximately follows the chi-squared distribution with two degrees of freedom. To account for multiple testing, associations with a false discovery rate (FDR) [41] < 0.05 were considered statistically significant and the corresponding CpGs were referred to as ‘identified CpGs’.

For identified CpGs, we investigated their associations with cumulative smoke exposure indicated by pack-years for ever smokers and with years since quitting for former smokers. Pack-years were calculated as the average number of cigarettes smoked per day divided by 20 and multiplied by the number of years smoked, and were log-transformed to be approximately normal distributed. Years since quitting were calculated as age at interview minus age at stopping smoking. The covariates adjusted and statistical inference were the same as those for smoking status, except that the model for pack-years was additionally adjusted for smoking status (former and current smokers) to investigate associations independent of smoking status.

### Replication of previously reported associations

After quality control, 18,671 CpGs reported from the largest meta-analysis performed by Joehanes et al. [11] were included in our study. For these CpGs, we investigated their associations with smoking status in our study. Given the sample size of our study and not to miss any potential replication, associations with a nominal *P* < 0.05 and the same direction as that reported by Joehanes et al. were considered to be replicated, and the corresponding CpGs were referred to as ‘replicated CpGs’.

### Familial confounding analysis

For the identified CpGs and replicated CpGs, we performed between- and within-sibship analyses [25, 42] to investigate if familial factors confound the associations. Given that never and former smokers had similar methylation levels for most of the CpGs, we combined them into one group. The new smoking status was thus analysed with current smokers as ‘1’ and the rest as ‘0’.

In the analysis, the methylation *M* values, smoking exposures and covariates were orthogonally transformed within sibships to obtain sibship means and within-sibship differences for these variables; see Stone et al. [42] for more details about the transformation. The between-sibship analyses investigated associations between sibship means for methylation levels and those for smoking exposures, and the within-sibship analyses investigated associations between within-sibship differences for methylation levels and those for smoking exposures. Associations estimated from the within-sibship analyses are independent of familial confounding, as the confounding effects of familial factors shared by siblings, both known and unknown, were cancelled out when using within-sibship differences. Evidence for familial confounding can be obtained by comparing between-sibship coefficient (*β*_B_) and within-sibship coefficient (*β*_W_). When β_B_ ≠ β_W_ and β_W_ ≈ 0, i.e. the association disappears when familial factors are adjusted, the observation is consistent with the association being due to familial confounding. When *β*_B_ ≈ *β*_W_ ≠ 0, i.e. the association is similar regardless of whether familial factors are adjusted, the observation is consistent with absence of evidence for familial confounding; see Carlin et al. [43] for more details about the implications from comparing β_B_ and β_W_.

### Causal inference analysis

We performed causal inference between smoking status and methylation using Inference about Causation through Examination of FAmiliaL CONfounding (ICE FALCON), a regression-based methodology for analysing twin data [44–48]. By causal is meant, that if it were possible to vary a predictor measure experimentally, the expected value of the outcome measure would change.

**Fig. 1:**
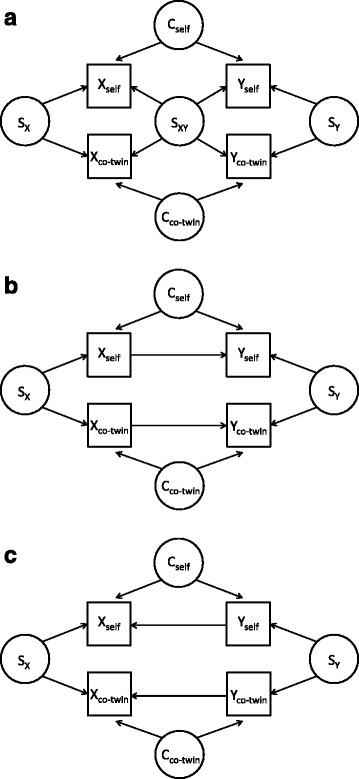
Some possible directed acyclic graphs for the cross-twin cross-trait correlation. **a** The cross-twin cross-trait correlation is due to familial confounding. **b** The cross-twin cross-trait correlation is due to the causal effect of *X* on *Y*. **c** The cross-twin cross-trait correlation is due to the causal effect of *Y* on *X*

As shown in Fig. 1, suppose there are two variables, *X* and *Y*, measured for pairs of twins, and for example, let *X* refer to smoking status and *Y* refer to methylation. Assume that *X* and *Y* are positively associated within an individual. Let *S* denote the unmeasured familial factors that affect both twins, *S*_X_ represents those factors that influence *X* values only, *S*_Y_ those that influence *Y* values only, and *S*_XY_ those that influence both *X* and *Y* values. For the purpose of explanation, let ‘self’ refer to an individual and ‘co-twin’ refer to the individual’s twin, but recognise that these labels can be exchanged and both twins within a pair are used in the analysis.

If there is a correlation between *Y*_self_ and *X*_co-twin_, it might be due to a familial confounder, *S*_XY_ (Fig. 1a). It could also be due to *X* having a causal effect on *Y* within an individual, provided *X*_self_ and *X*_co-twin_ are correlated (Fig. 1b), or to *Y* having a casual effect on *X*, provided *Y*_self_ and *Y*_co-twin_ are correlated (Fig. 1c). Note that the confounders specific to an individual, *C*_self_ and *C*_co-twin_, do not of themselves result in a correlation between *Y*_self_ and *X*_co-twin_.

Using the Generalised Estimating Equations (GEE), fitted using the *geeglm()* function from R package *geepack* [49], to take into account any correlation in Y between twins within the same pair, three models are fitted:

Model 1: *E*(*Y*_self_) = *α* + *β*_self_*X*_self_

Model 2: *E*(*Y*_self_) = *α* + *β*_co-twin_*X*_co-twin_

Model 3: *E*(*Y*_self_) = *α* + *β*′_self_*X*_self_ + *β*′_co-twin_*X*_co-twin_

If the correlation between *Y*_self_ and *X*_co-twin_ is solely due to familial confounders (Fig. 1a), the marginal association between *Y*_self_ and *X*_self_ (*β*_self_ in model 1) and the marginal association between *Y*_self_ and *X*_co-twin_ (*β*_co-twin_ in model 2) must both be non-zero. Adjusting for *X*_self_, however, the conditional association between *Y*_self_ and *X*_co-twin_ (*β*′_co-twin_ in model 3) is expected to attenuate from *β*_co-twin_ in model 2 towards the null. Similarly, adjusting for *X*_co-twin_ (model 3), the conditional association between *Y*_self_ and *X*_self_ (*β*′_self_ in model 3) is expected to attenuate from *β*_self_ in model 1 towards the null.

If the correlation between *Y*_self_ and *X*_co-twin_ is solely due to a causal effect from *X* to *Y* (Fig. 1b), *Y*_self_ and *X*_co-twin_ in model 2 will be associated through two pathways: the confounder *S*_X_, and conditioning on the collider *Y*_co-twin_ (GEE analysis in effect conditions on *Y*_co-twin_). Conditioning on *Y*_co-twin_ induces a negative correlation between *X*_co-twin_ and *Y*_self_ (note that we assume *X* and *Y* are positively associated within an individual), so that *β*_co-twin_ in model 2 depends on the within-pair correlations in *X* (ρ_X_) and in *Y* (ρ_Y_): if ρ_X_ > ρ_Y_, *β*_co-twin_ is expected to be positive; otherwise *β*_co-twin_ to be negative. Conditioning on *X*_self_ (model 3), both pathways are blocked and the conditional association (*β*′_co-twin_ in model 3) is expected to attenuate towards the null.

If the correlation between *Y*_self_ and *X*_co-twin_ is solely due to a causal effect from *Y* to *X* (Fig. 1c), in model 2 the pathway through *S*_X_ is blocked due to *X*_self_ as a collider, and the pathway through *S*_Y_ is blocked due to that GEE analysis in effect conditions on *Y*_co-twin_, so there is no marginal association between *Y*_self_ and *X*_co-twin_, and *β*_co-twin_ of model 2 is expected to be zero.

We studied methylation at the identified CpGs and replicated CpGs, respectively. For each group of CpGs, methylation was analysed as a weighted methylation score, calculated as the sum of the products of methylation level and weight of each CpG. For a locus containing multiple CpGs, only the CpG with the smallest *P* value was included in the methylation score. For the identified CpGs, the methylation level was the standardised *M* value and the weight was the log odds ratio for smoking status. For the replicated CpGs, the methylation level was the Beta value, the scale used in the meta-analysis, and the weight was the *Z* statistic reported by Joehanes et al. [11]. Smoking status was analysed as a binary variable with current smokers as ‘1’ and the rest as ‘0’. We first used smoking status to be *X* and methylation score to be *Y* and regressed methylation score on smoking status. We then exchanged *X* and *Y* to regress smoking status on methylation score and undertook the same analyses. The data for 132 twin pairs were used. We made statistical inference about the change in regression coefficient using one-sided *t* test with a standard error computed using nonparametric bootstrap method. That is, twin pairs were randomly sampled with replacement to generate 1000 new datasets with the same sample size as the original dataset. ICE FALCON was then applied to each dataset to calculate the change in regression coefficient for that dataset and standard error was then estimated by computing the standard deviation.

## Results

### Characteristics of the sample

The mean (standard deviation [SD]) age for the 479 women was 56.4 (7.9) years. The women included 291 (60.8%) never smokers, 147 (30.7%) former smokers and 41 (8.5%) current smokers. Ever smokers had a median (interquartile range) of 7.0 (13.8) pack-years. Former smokers had an average (SD) of 21.5 (11.4) years since quitting.

### Epigenome-wide analysis results

**Fig. 2:**
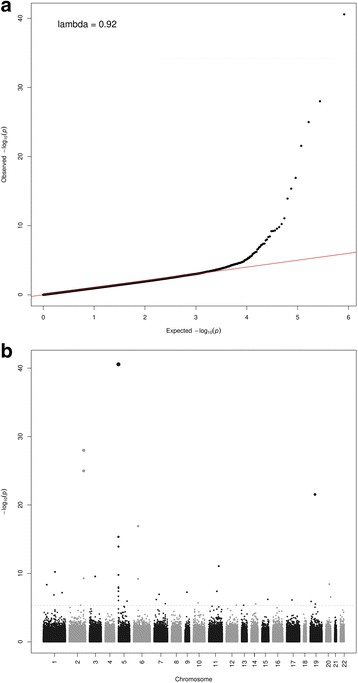
Q-Q plot (**a**) and Manhattan plot (**b**) for the results from the epigenome-wide association analysis between DNA methylation and smoking status

Methylation at 39 CpGs located at 27 loci was found to be associated with smoking status (Table 1; Q-Q plot and Manhattan plot in Fig. 2). Associations for 37 of the 39 CpGs have been reported by at least two studies and associations for two CpGs, cg06226150 (*SLC2A4RG*) and cg21733098 (*12q24.32*), have only been reported from the meta-analysis performed by Joehanes et al. [11]. For all 39 CpGs, current smokers had the lowest methylation level (Table 1). The 27 loci included several consistently reported loci, such as *AHRR* (9 CpGs), *2q37.1* (3 CpGs), *6p21.33* (3 CpGs), and *F2RL3* (1 CpG).

**Table 1:** 39 CpGs at which methylation was found to be associated with smoking status with FDR < 0.05

CpG	CHR	Loci	Methylation level, mean (standard deviation)			*P*	FDR
			Never smokers	Former smokers	Current smokers		
cg05575921	5	AHRR	0.82 (0.04)	0.79 (0.05)	0.69 (0.08)	2.69E-41	1.11E-35
cg05951221	2	2q37.1	0.48 (0.05)	0.44 (0.06)	0.38 (0.06)	1.01E-28	2.08E-23
cg01940273	2	2q37.1	0.69 (0.04)	0.66 (0.05)	0.60 (0.05)	1.03E-25	1.41E-20
cg03636183	19	F2RL3	0.72 (0.04)	0.70 (0.05)	0.64 (0.06)	2.86E-22	2.94E-17
cg06126421	6	6p21.33	0.79 (0.05)	0.76 (0.06)	0.72 (0.06)	1.22E-17	1.00E-12
cg26703534	5	AHRR	0.68 (0.03)	0.69 (0.03)	0.64 (0.03)	4.44E-16	3.04E-11
cg21161138	5	AHRR	0.77 (0.03)	0.76 (0.04)	0.72 (0.05)	1.21E-14	7.11E-10
cg11660018	11	PRSS23	0.59 (0.04)	0.57 (0.04)	0.54 (0.04)	8.59E-12	4.42E-07
cg09935388	1	GFI1	0.82 (0.05)	0.81 (0.05)	0.75 (0.07)	5.90E-11	2.70E-06
cg25648203	5	AHRR	0.84 (0.02)	0.83 (0.02)	0.81 (0.03)	1.63E-10	6.71E-06
cg19859270	3	GPR15	0.93 (0.01)	0.93 (0.01)	0.92 (0.01)	2.77E-10	1.04E-05
cg03329539	2	2q37.1	0.47 (0.05)	0.46 (0.05)	0.42 (0.04)	5.04E-10	1.73E-05
cg24859433	6	6p21.33	0.88 (0.02)	0.88 (0.02)	0.86 (0.02)	6.02E-10	1.85E-05
cg14753356	6	6p21.33	0.47 (0.06)	0.45 (0.06)	0.43 (0.05)	6.28E-10	1.85E-05
cg07339236	20	ATP9A	0.17 (0.04)	0.16 (0.04)	0.13 (0.03)	3.68E-09	1.01E-04
cg04885881	1	1p36.22	0.48 (0.05)	0.47 (0.05)	0.44 (0.05)	4.46E-09	1.15E-04
cg23916896	5	AHRR	0.29 (0.07)	0.27 (0.06)	0.23 (0.06)	1.01E-08	2.43E-04
cg14817490	5	AHRR	0.30 (0.04)	0.03 (0.04)	0.26 (0.04)	1.37E-08	3.14E-04
cg11902777	5	AHRR	0.08 (0.02)	0.08 (0.02)	0.06 (0.02)	4.01E-08	8.55E-04
cg21611682	11	LRP5	0.61 (0.03)	0.60 (0.03)	0.58 (0.03)	4.16E-08	8.55E-04
cg01692968	9	9q31.1	0.41 (0.05)	0.39 (0.05)	0.38 (0.05)	5.57E-08	1.09E-03
cg08709672	1	AVPR1B	0.60 (0.03)	0.59 (0.03)	0.57 (0.03)	6.54E-08	1.22E-03
cg07826859	7	MYO1G	0.66 (0.04)	0.65 (0.04)	0.63 (0.03)	1.14E-07	2.04E-03
cg25189904	1	GNG12	0.53 (0.06)	0.51 (0.07)	0.47 (0.07)	1.36E-07	2.33E-03
cg17287155	5	AHRR	0.86 (0.03)	0.85 (0.03)	0.84 (0.03)	2.19E-07	3.61E-03
cg06226150	20	SLC2A4RG	0.28 (0.03)	0.28 (0.02)	0.26 (0.02)	2.85E-07	4.51E-03
cg23161492	15	ANPEP	0.30 (0.05)	0.29 (0.05)	0.26 (0.05)	6.19E-07	9.43E-03
cg09022230	7	TNRC18	0.76 (0.04)	0.75 (0.04)	0.73 (0.04)	6.57E-07	9.65E-03
cg19572487	17	RARA	0.63 (0.05)	0.61 (0.05)	0.60 (0.06)	7.54E-07	1.07E-02
cg03991871	5	AHRR	0.89 (0.03)	0.89 (0.03)	0.86 (0.04)	9.13E-07	1.25E-02
cg14580211	5	C5orf62	0.76 (0.04)	0.75 (0.04)	0.73 (0.04)	1.12E-06	1.48E-02
cg15187398	19	MOBKL2A	0.53 (0.05)	0.51 (0.05)	0.49 (0.04)	1.25E-06	1.60E-02
cg10750182	10	C10orf105	0.62 (0.03)	0.62 (0.03)	0.60 (0.03)	2.03E-06	2.53E-02
cg25949550	7	CNTNAP2	0.13 (0.02)	0.13 (0.02)	0.12 (0.02)	2.64E-06	3.19E-02
cg05284742	14	ITPK1	0.78 (0.03)	0.77 (0.03)	0.76 (0.04)	2.76E-06	3.24E-02
cg23931381	19	ARRDC2	0.89 (0.02)	0.88 (0.02)	0.87 (0.02)	2.98E-06	3.40E-02
cg26271591	2	NFE2L2	0.46 (0.06)	0.45 (0.06)	0.41 (0.06)	4.40E-06	4.72E-02
cg03646329	13	LPAR6	0.82 (0.04)	0.81 (0.05)	0.79 (0.05)	4.47E-06	4.72E-02
cg21733098	12	12q24.32	0.76 (0.06)	0.75 (0.07)	0.72 (0.06)	4.47E-06	4.72E-02

Of the 39 CpGs and at a 5% FDR, methylation at 18 CpGs was negatively associated with pack-years and at 20 CpGs was positively associated with years since quitting. Methylation at 15 CpGs was associated with pack-years and years since quitting both (Table 2).

**Table 2:** Associations of methylation at the 39 identified CpGs with pack-years and years since quitting

CpG	Pack-years			Years since quitting		
	Estimate (SE)	*P* value	FDR	Estimate (SE)	*P* value	FDR
cg05575921	− 10.68 (2.18)	1.11E-06	1.44E-05	1.63 (0.30)	1.11E-35	7.35E-08
cg05951221	− 10.32 (1.70)	3.04E-09	1.19E-07	1.66 (0.25)	2.08E-23	1.55E-10
cg01940273	− 8.99 (1.58)	1.93E-08	3.76E-07	1.47 (0.23)	1.41E-20	1.15E-09
cg03636183	− 4.65 (1.70)	5.35E-03	1.90E-02	0.98 (0.24)	2.94E-17	5.87E-05
cg06126421	− 5.92 (2.20)	6.15E-03	2.00E-02	1.31 (0.32)	1.00E-12	3.36E-05
cg26703534	0.28 (0.98)	8.02E-01	8.02E-01	− 0.3 (0.15)	3.04E-11	3.44E-02
cg21161138	− 4.30 (1.62)	6.89E-03	2.07E-02	0.65 (0.24)	7.11E-10	5.32E-03
cg11660018	− 3.74 (1.13)	7.82E-04	4.36E-03	0.83 (0.17)	4.42E-07	5.86E-07
cg09935388	− 6.87 (2.40)	3.06E-03	1.19E-02	1.10 (0.35)	2.70E-06	1.63E-03
cg25648203	− 2.25 (1.28)	7.19E-02	1.27E-01	0.37 (0.19)	6.71E-06	4.18E-02
cg19859270	− 2.69 (1.38)	4.65E-02	9.55E-02	0.58 (0.21)	1.04E-05	4.41E-03
cg03329539	− 6.27 (1.35)	3.59E-06	3.50E-05	0.83 (0.21)	1.73E-05	5.13E-05
cg24859433	− 1.45 (1.21)	2.04E-01	2.65E-01	0.41 (0.18)	1.85E-05	1.78E-02
cg14753356	− 2.64 (1.12)	1.63E-02	3.74E-02	0.38 (0.18)	1.85E-05	2.28E-02
cg07339236	− 2.71 (1.93)	1.51E-01	2.26E-01	0.89 (0.30)	1.01E-04	2.21E-03
cg04885881	− 2.16 (1.29)	7.80E-02	1.32E-01	0.28 (0.20)	1.15E-04	1.18E-01
cg23916896	− 4.32 (2.41)	6.61E-02	1.23E-01	0.47 (0.37)	2.43E-04	1.84E-01
cg14817490	− 3.31 (1.35)	1.26E-02	3.28E-02	0.35 (0.21)	3.14E-04	1.02E-01
cg11902777	− 7.19 (2.15)	6.99E-04	4.36E-03	0.65 (0.32)	8.55E-04	4.06E-02
cg21611682	− 2.04 (0.80)	9.75E-03	2.72E-02	0.27 (0.12)	8.55E-04	2.25E-02
cg01692968	− 2.37 (1.42)	8.83E-02	1.43E-01	0.72 (0.21)	1.09E-03	5.77E-04
cg08709672	− 1.05 (0.80)	1.75E-01	2.43E-01	0.29 (0.12)	1.22E-03	1.91E-02
cg07826859	− 1.75 (0.97)	6.23E-02	1.21E-01	0.17 (0.15)	2.04E-03	2.48E-01
cg25189904	− 7.02 (2.23)	1.38E-03	6.72E-03	0.74 (0.34)	2.33E-03	2.45E-02
cg17287155	− 1.67 (1.27)	1.74E-01	2.43E-01	0.31 (0.18)	3.61E-03	7.50E-02
cg06226150	− 1.37 (0.88)	1.12E-01	1.74E-01	0.21 (0.14)	4.51E-03	1.24E-01
cg23161492	− 5.41 (1.57)	5.18E-04	4.04E-03	0.68 (0.24)	9.43E-03	3.95E-03
cg09022230	0.73 (1.23)	5.42E-01	6.21E-01	0.15 (0.19)	9.65E-03	4.52E-01
cg19572487	− 4.19 (1.44)	2.96E-03	1.19E-02	0.60 (0.20)	1.07E-02	2.64E-03
cg03991871	− 5.59 (2.37)	1.63E-02	3.74E-02	0.40 (0.36)	1.25E-02	2.48E-01
cg14580211	− 0.43 (1.40)	7.46E-01	7.86E-01	0.43 (0.21)	1.48E-02	3.25E-02
cg15187398	− 1.28 (1.31)	3.10E-01	3.90E-01	0.15 (0.20)	1.60E-02	4.27E-01
cg10750182	− 0.64 (0.67)	3.20E-01	3.90E-01	0.15 (0.10)	2.53E-02	1.05E-01
cg25949550	− 2.80 (1.25)	2.24E-02	4.86E-02	0.59 (0.20)	3.19E-02	2.17E-03
cg05284742	− 0.49 (1.13)	6.58E-01	7.13E-01	0.21 (0.16)	3.24E-02	1.81E-01
cg23931381	0.71 (1.50)	6.03E-01	6.72E-01	0.20 (0.23)	3.40E-02	4.26E-01
cg26271591	− 0.44 (1.74)	7.87E-01	8.02E-01	0.34 (0.26)	4.72E-02	1.77E-01
cg03646329	− 2.47 (1.98)	1.99E-01	2.65E-01	0.63 (0.30)	4.72E-02	3.08E-02
cg21733098	− 1.78 (2.49)	4.55E-01	5.38E-01	− 0.03 (0.38)	4.72E-02	9.23E-01

Regression coefficients were reported as being multiplied by 100, as well as for standard errors

### Replication for previously reported associations

For the associations for 18,671 CpGs reported by Joehanes et al. [11], 1882 were replicated with a nominal *P* < 0.05 and in the same direction, and the 133 most significant associations also had a FDR < 0.05. Of the 1882 replications, 1154 were for the novel CpGs reported by Joehanes et al. (Additional file 1: Table S1).

### Between- and within-sibship analyses results

For the 39 identified CpGs, no evidence for a difference between *β*_B_ and *β*_W_ was found for any CpG (Table 3; all *P* values > 0.05 from the *β*_B_ and *β*_W_ comparison). The same results were found from the analyses of pack-years and years since quitting (Table 3).

**Table 3:** Associations of methylation at the 39 identified CpGs with smoking status, pack-years and years since quitting from the between- and within-sibship analyses

CpG	Smoking status			Pack-years			Years since quitting		
	Between-sibship coefficient (SE)	Within-sibship coefficient (SE)	P*	Between-sibship coefficient (SE)	Within-sibship coefficient (SE)	P*	Between-sibship coefficient (SE)	Within-sibship coefficient (SE)	P*
cg05575921	− 0.87 (0.12)	− 0.93 (0.08)	0.65	− 14.53 (4.65)	− 6.58 (3.76)	0.18	1.53 (0.57)	1.76 (0.52)	0.77
cg05951221	− 0.59 (0.10)	− 0.47 (0.07)	0.32	− 13.28 (4.08)	− 6.74 (2.87)	0.19	1.75 (0.43)	1.56 (0.41)	0.75
cg01940273	− 0.60 (0.10)	− 0.47 (0.06)	0.26	− 8.04 (3.30)	− 6.32 (2.88)	0.69	1.36 (0.35)	1.77 (0.41)	0.44
cg03636183	− 0.56 (0.09)	− 0.41 (0.06)	0.18	− 2.63 (4.55)	− 2.93 (2.90)	0.96	0.57 (0.50)	1.18 (0.41)	0.34
cg06126421	− 0.34 (0.14)	− 0.48 (0.08)	0.37	− 13.31 (6.51)	− 5.02 (3.69)	0.27	1.59 (0.68)	1.62 (0.52)	0.98
cg26703534	− 0.21 (0.06)	− 0.30 (0.04)	0.17	− 2.34 (2.07)	2.74 (1.62)	0.05	− 0.33 (0.28)	− 0.41 (0.24)	0.84
cg21161138	− 0.36 (0.09)	− 0.37 (0.06)	0.91	− 4.37 (4.08)	− 3.20 (2.41)	0.81	0.46 (0.47)	0.91 (0.36)	0.45
cg11660018	− 0.26 (0.08)	− 0.20 (0.04)	0.46	− 5.50 (2.48)	0.51 (2.11)	0.07	1.01 (0.28)	0.88 (0.29)	0.75
cg09935388	− 0.58 (0.14)	− 0.53 (0.10)	0.77	− 2.29 (5.39)	− 5.44 (4.01)	0.64	0.71 (0.68)	1.32 (0.59)	0.50
cg25648203	− 0.16 (0.08)	− 0.31 (0.05)	0.10	− 6.26 (2.96)	− 1.05 (2.12)	0.15	0.77 (0.36)	0.27 (0.30)	0.29
cg19859270	− 0.28 (0.08)	− 0.22 (0.05)	0.51	− 4.06 (3.48)	− 1.60 (2.63)	0.57	0.70 (0.34)	0.39 (0.38)	0.54
cg03329539	− 0.31 (0.08)	− 0.28 (0.06)	0.79	− 4.34 (3.26)	− 6.70 (2.45)	0.56	0.78 (0.35)	0.95 (0.35)	0.73
cg24859433	− 0.15 (0.07)	− 0.24 (0.05)	0.25	− 3.51 (2.81)	− 0.74 (2.12)	0.43	0.71 (0.30)	0.47 (0.32)	0.59
cg14753356	− 0.13 (0.07)	− 0.16 (0.04)	0.73	− 0.21 (3.36)	− 3.43 (1.78)	0.40	0.61 (0.34)	0.33 (0.27)	0.52
cg07339236	− 0.29 (0.11)	− 0.32 (0.07)	0.83	− 1.48 (4.44)	− 0.56 (3.24)	0.87	0.88 (0.48)	0.62 (0.48)	0.70
cg04885881	− 0.26 (0.08)	− 0.24 (0.05)	0.80	− 0.68 (2.88)	0.24 (2.12)	0.80	0.45 (0.32)	0.26 (0.32)	0.67
cg23916896	− 0.40 (0.15)	− 0.49 (0.10)	0.60	− 9.52 (5.34)	8.79 (4.31)	0.01	0.72 (0.62)	− 0.13 (0.67)	0.35
cg14817490	− 0.18 (0.09)	− 0.28 (0.05)	0.32	− 2.65 (3.72)	− 2.45 (2.15)	0.96	0.30 (0.42)	0.55 (0.30)	0.63
cg11902777	− 0.40 (0.14)	− 0.44 (0.09)	0.80	− 14.37 (4.30)	− 2.35 (3.70)	0.03	1.22 (0.54)	0.70 (0.56)	0.50
cg21611682	− 0.15 (0.05)	− 0.16 (0.03)	0.88	− 1.63 (1.75)	− 1.82 (1.48)	0.93	0.34 (0.22)	0.42 (0.22)	0.80
cg01692968	− 0.11 (0.09)	− 0.16 (0.06)	0.61	− 4.60 (3.02)	0.19 (2.32)	0.21	0.76 (0.39)	0.51 (0.34)	0.63
cg08709672	− 0.07 (0.06)	− 0.17 (0.03)	0.12	1.06 (2.28)	− 1.11 (1.30)	0.41	0.08 (0.26)	0.56 (0.19)	0.13
cg07826859	− 0.16 (0.06)	− 0.20 (0.04)	0.52	− 2.02 (2.59)	− 1.05 (1.69)	0.75	0.09 (0.26)	0.41 (0.24)	0.37
cg25189904	− 0.46 (0.11)	− 0.29 (0.09)	0.21	− 11.29 (4.40)	− 0.82 (4.21)	0.09	0.20 (0.57)	1.09 (0.61)	0.29
cg17287155	− 0.23 (0.08)	− 0.19 (0.05)	0.65	− 1.78 (2.87)	2.63 (2.03)	0.21	0.49 (0.3)	− 0.16 (0.32)	0.14
cg06226150	− 0.19 (0.05)	− 0.13 (0.04)	0.36	− 2.55 (2.48)	− 2.00 (1.44)	0.85	− 0.14 (0.26)	0.36 (0.22)	0.14
cg23161492	− 0.28 (0.11)	− 0.24 (0.06)	0.74	− 8.68 (4.48)	− 4.66 (2.44)	0.43	0.55 (0.51)	0.81 (0.37)	0.68
cg09022230	− 0.12 (0.08)	− 0.25 (0.05)	0.17	5.91 (2.41)	− 3.44 (1.92)	0.00	− 0.13 (0.34)	0.73 (0.29)	0.06
cg19572487	− 0.14 (0.07)	− 0.20 (0.06)	0.54	− 8.14 (3.10)	− 1.86 (2.16)	0.10	0.75 (0.37)	0.69 (0.33)	0.91
cg03991871	− 0.39 (0.16)	− 0.38 (0.08)	0.98	− 5.86 (5.57)	− 0.98 (4.13)	0.48	0.10 (0.63)	0.29 (0.63)	0.83
cg14580211	− 0.15 (0.09)	− 0.24 (0.05)	0.33	− 3.40 (3.54)	0.79 (2.41)	0.33	1.00 (0.37)	0.46 (0.33)	0.27
cg15187398	− 0.18 (0.08)	− 0.18 (0.05)	0.96	− 2.81 (3.07)	1.55 (2.24)	0.25	0.23 (0.39)	0.15 (0.35)	0.87
cg10750182	− 0.08 (0.04)	− 0.11 (0.03)	0.53	− 0.79 (1.75)	0.47 (1.18)	0.55	0.07 (0.19)	0.23 (0.17)	0.55
cg25949550	− 0.13 (0.07)	− 0.20 (0.05)	0.39	− 1.05 (2.47)	0.12 (2.36)	0.73	0.44 (0.30)	0.81 (0.34)	0.41
cg05284742	− 0.16 (0.06)	− 0.14 (0.05)	0.86	3.53 (2.18)	− 1.18 (1.94)	0.11	0.20 (0.30)	0.30 (0.28)	0.81
cg23931381	− 0.08 (0.08)	− 0.20 (0.06)	0.25	1.09 (3.58)	1.77 (2.65)	0.88	− 0.16 (0.43)	0.46 (0.37)	0.27
cg26271591	− 0.16 (0.10)	− 0.32 (0.07)	0.19	− 4.11 (4.56)	0.95 (3.10)	0.36	0.41 (0.46)	0.52 (0.45)	0.87
cg03646329	− 0.29 (0.13)	− 0.27 (0.08)	0.90	− 10.07 (5.29)	− 1.22 (3.22)	0.15	0.80 (0.64)	0.66 (0.46)	0.86
cg21733098	− 0.37 (0.16)	− 0.31 (0.09)	0.76	− 4.20 (6.47)	2.68 (4.37)	0.38	0.19 (0.70)	− 0.14 (0.67)	0.74

Regression coefficients from the analyses for pack-years and years since quitting were reported as being multiplied by 100, as well as for standard errors

*P-value from comparing the between-sibship coefficient with the within-sibship coefficient

For the 1882 replicated CpGs, no evidence for a difference between *β*_B_ and *β*_W_ was found for any CpG (Additional file 2: Table S2; the smallest *P* value = 1.3 × 10^− 3^ and the smallest FDR = 0.99 from the *β*_*B*_ and *β*_W_ comparison).

### ICE FALCON analysis results

Within twin pairs, the correlation in smoking status was 0.11 (95% confidence interval (CI) − 0.06, 0.27), smaller than the correlations in methylation scores for the replicated CpGs and for the identified CpGs, which were 0.37 (95% CI 0.23, 0.50) and 0.22 (95% CI 0.05, 0.37), respectively.

The ICE FALCON results for methylation at the replicated CpGs are shown in Table 4. From the analysis in which smoking status was the predictor and methylation score the outcome, a women’s methylation score was associated with her own smoking status (model 1; *β*_self_ = 74.6, 95% CI 55.3, 93.9), and negatively associated with her co-twin’s smoking status (model 2; *β*_co-twin_ = − 30.8, 95% CI − 57.7, − 4.0). Conditioning on her co-twin’s smoking status (model 3), *β*′_self_ remained unchanged (*P* = 0.41) compared with *β*_self_ in model 1, while conditioning on her own smoking status (model 3), *β*_co-twin_ in model 2 attenuated by 123.3% (95% CI 49.6%, 185.2%; *P* = 0.002) to be *β*′_co-twin_ of 2.5 (95% CI − 16.3, 21.3). From the analysis in which methylation score was the predictor and smoking status the outcome, a woman’s smoking status was associated with her own methylation score (model 1; *β*_self_ = 4.1, 95% CI 2.7, 5.4), but not with her co-twin’s methylation score (model 2; *β*_co-twin_ = 0.4, 95% CI − 1.0, 1.8). In model 3, *β*′_self_ and *β*′_co-twin_ remained unchanged (both *P* > 0.1) compared with *β*_self_ in model 1 and *β*_co-twin_ in model 2, respectively. These results were consistent with that smoking has a causal effect on the overall methylation level at these CpGs, but not in the opposite direction. Similar results were found and a similar causality was inferred for smoking status and the overall methylation level at the identified CpGs (Table 4).

**Table 4:** Results from the ICE FALCON analyses

CpGs	Coefficient	Model 1		Model 2		Model 3		Change	
		Estimate (SE)	*P*	Estimate (SE)	*P*	Estimate (SE)	*P*	Estimate (SE)	*P*
CpGs reported by Joehanes et al.									
Smoking as the predictor	*β*_self_	74.61 (9.87)	4.0E-14	–	–	75.45 (9.29)	4.4E-16	0.84 (3.60)	4.1E-01
	*β*_co-twin_	–	–	− 30.84 (13.69)	2.4E-02	2.50 (9.57)	7.9E-01	− 33.34 (11.60)	2.1E-03
Methylation score as the predictor	*β*_self_	4.07 (0.70)	7.5E-09	–	–	4.45 (0.81)	3.6E-08	0.39 (0.47)	2.1E-01
	*β*_co-twin_	–	–	0.41 (0.72)	5.7E-01	− 1.00 (0.82)	2.2E-01	− 1.42 (1.15)	1.1E-01
CpGs identified from our study									
Smoking as the predictor	*β*_self_	27.70 (3.65)	3.4E-14	–	–	26.89 (3.79)	1.2E-12	− 0.81 (0.89)	1.8E-01
	*β*_co-twin_	–	–	− 12.36 (3.86)	1.4E-03	− 3.45 (2.58)	1.8E-01	− 8.90 (5.52)	5.3E-02
Methylation score as the predictor	*β*_self_	10.24 (2.19)	1.3E-08	–	–	11.14 (2.47)	6.7E-06	0.90 (1.27)	2.4E-01
	*β*_co-twin_	–	–	− 4.48 (2.65)	9.2E-02	− 3.86 (2.66)	1.5E-01	0.61 (3.77)	4.4E-01

Regression coefficients from the analyses in which the methylation score as the predictor were reported as being multiplied by 100, as well as for standard errors

## Discussion

We performed an EWAS of smoking for a sample of middle-aged women and found 39 CpGs at which methylation was associated with smoking status. Our study confirmed the associations for several previously consistently reported loci including *AHRR*, *F2RL3*, *2q37.1*, and *6p21.33*, and for two novel CpGs, cg06226150 (*SLC2A4RG*) and cg21733098 (*12q24.32*), reported by the largest meta-analysis [11] so far. In addition, we replicated the associations for 1882 CpGs reported by the meta-analysis. The investigation of causation suggests that smoking has a causal effect on DNA methylation, not vice versa or being due to familial confounding.

To the best of our knowledge, our study is the first study to confirm the associations for cg06226150 and cg21733098. cg06226150 is located at the promoter of, and potentially regulates the expression of, *SLC2A4RG* (solute carrier family 2 member 4 regulator gene). *SLC2A4RG* is involved in the Gene Ontology pathway for regulation of transcription (GO:0006355). Protein encoded by *SLC2A4RG* regulates the activation of *SLC2A4* (solute carrier family 2 member 4). *SLC2A4* is involved in the glucose transportation across cell membranes stimulated by insulin. Genetic variants at *SLC2A4RG* have been found to be associated with inflammatory bowel disease [50] and prostate cancer [51]. cg21733098 is located at an intergenic region on *12q24.32*. The region contains several long non-coding RNA genes. Little is known about the regulatory function of cg21733098. The biological relevance of smoking to blood methylation at these two CpGs is largely unknown, and more research are warranted.

We found evidence that 18 and 20 of the identified CpGs were also associated with pack-years and years since quitting, respectively. Given that smokers have lower methylation levels at the identified CpGs, the negative associations with pack-years imply that there appear to be dose-relationships between smoking and methylation at the 18 CpGs, and the positive associations with years quitting smoking imply that methylation changes at the 20 CpGs tend to reverse after cessation. The dose-relationship and reversion have also been reported by several studies [4, 9–12, 15, 16, 19, 20, 22].

Our study, as one of the first studies, provides insights into the causality underlying the cross-sectional association between smoking and blood DNA methylation. Our results are inconsistent with the proposition that the cross-sectional association is due to familial confounding, e.g. shared genes and/or environment. The roles of shared genes and/or environment are also in part unsupported by that certain smoking-related loci, such as *AHRR* and *F2RL3*, are observed across Europeans [3, 5, 8–11, 16, 19, 20, 22], South Asians [8], Arabian Asians [21], East Asians [12, 23], and African Americans [7, 11, 13, 18], who have different germline genetic backgrounds and environments. Our results support that smoking has a causal effect on the overall methylation at the identified CpGs and at the replicated CpGs, but not vice versa. Results from the two-step MR analysis performed by Jhun et al. [31] also suggest that differential methylation at cg03636183 (*F2RL3*) and cg19859270 (*GPR15*) between current and never smokers are consequential to smoking under the assumptions of MR.

That smoking causes changes in methylation is also supported to some extent by other evidence. The ‘reversion’ phenomenon is in line with the ‘experimental evidence’ criterion proposed by Bradford Hill, i.e. ‘reducing or eliminating a putatively harmful exposure and seeing if the frequency of disease subsequently declines’ [52]. The associations between cord blood methylation for newborns at some active-smoking-related loci, such as *AHRR* and *GFI1*, and maternal smoking in pregnancy [53] also imply that smoking is likely to cause methylation changes at these loci. Additionally, some smoking-related loci are involved in the metabolism of smoking-released chemicals. *AHRR* gene encodes a repressor of the aryl hydrocarbon receptor (*AHR*) gene, the protein encoded by which is involved in the regulation of biological response to planar aromatic hydrocarbons. Polycyclic aromatic hydrocarbons, one main smoking-related toxic and carcinogenic substance, trigger AHR signalling cascade [16, 22]. Protein coded by the *AHR* gene activates the expression of the *AHRR* gene, which in turn represses the function of AHR through a negative feedback mechanism [54]. That hypomethylation at *AHRR* gene caused by smoking is biologically plausible.

That smoking causes changes in blood methylation has great clinical and etiological implications: methylation might mediate the effects of smoking on smoking-related health outcomes. As introduced above, there have been a few studies [26–29] investigating the mediating role of methylation. A better understanding of the mechanisms of smoking affecting health is expected with more investigations on methylation.

Our study shows the value of ICE FALCON in causality assessment for observational associations. Associations from observational studies can be due to confounding and, although analyses of measured potential confounders can eliminate some confounding, there is always the possibility of unmeasured confounding, even with prospective studies. With recent discoveries of genetic markers that predict variation in risk factors, the MR concept has been explored by epidemiologists. MR uses measured genetic variants as the instrumental variable and the results of MR might be biased due to several factors such as strengthen of instrumental variable, directional pleiotropy, and unmeasured confounding [55]. ICE FALCON is a novel approach to making inference about causation. It in effect uses the familial causes of exposure and of outcome as instrumental variables. The familial causes are not measured but surrogated by co-twin’s measured exposure and outcome. Thus, ICE FALCON resembles a bidirectional MR approach [56]. The instrumental variables consider all familial causes in exposure and in outcome, thus potentially less biased by their strengths than a finite number of genetic markers. More importantly, even should directional pleiotropy exist, the attenuation in the coefficient for co-twin’s exposure after adjusting for an individual’s own exposure also supports a causal effect.

## Conclusions

We found evidence that in the peripheral blood from middle-aged women, DNA methylation at several loci is associated with smoking. By investigating causation underlying the association, our study found evidence consistent with smoking having a causal effect on methylation, but not vice versa.

## Additional files

Additional file 1: **Table S1.** This file includes **Table S1**: Associations for the 1882 replicated CpGs. (XLSX 156 kb)

Additional file 2: **Table S2.** This file includes **Table S2**: Associations of methylation at the 1882 replicated CpGs with smoking status from the between- and within-sibship analyses. (XLSX 100 kb)

## Abbreviations

AHRR: Aryl hydrocarbon receptor repressor gene
CI: Confidence interval
CpG: Cytosine-guanine dinucleotide
EWAS: Epigenome-wide association study
F2RL3: F2R-like thrombin or trypsin receptor 3 gene
FDR: False discovery rate
GEE: Generalised estimating equations
GFI1: Growth factor independent 1 transcriptional repressor gene
GPR15: G protein-coupled receptor 15 gene
ICE FALCON: Inference about causation through examination of familial confounding
MR: Mendelian randomisation
SD: standard deviation
SLC2A4RG: Solute carrier family 2 member 4 regulator gene
